# Significant association of serum *H. pylori* IgG antibody titer with kidney function in renal transplanted patients

**DOI:** 10.12861/jrip.2013.08

**Published:** 2013-03-01

**Authors:** Hamid Nasri, Mahmoud Rafieian-Kopaei

**Affiliations:** ^1^Department of Internal Medicine, Shahrekord University of Medical Sciences, Shahrekord, Iran; ^2^Medical Plants Research Center, Shahrekord University of Medical Sciences, Shahrekord, Iran

**Keywords:** Kidney transplant, *Helicobacter pylori*, Renal function

## Abstract

**Introduction:** There is a paucity of information about the *Helicobacter pylori* (*H.Pylori*) infection in kidney recipients.

**Objectives:** At this study, we sought to investigate the association of some demographic and biochemical indices of kidney transplant patients with serum *H. pylori* antibody value.Patients and Methods: A total of 72 patients were enrolled in this study.

** Results:** The mean of serum *H. pylori* IgG antibody titer was 3 ±4.6 U/mL (median= 1 U/mL). A significant positive correlation between serum *H. pylori* IgG antibody level and creatinine clearance was seen (r = 0.26, P = 0.02).

**Conclusion:** The important finding of this study was the significant positive association of serum *H. pylori* IgG antibody titer with renal function in renal transplant patient. It means that with gradual loss of kidney function, the probability of *H. pylori*-infection is increased. Hence, *H. pylori* infection which is one of the most important and treatable factor leading to a peptic ulcer disease should be treated and eradicated before embarking for transplant.

**Implication for health policy/practice/research/medical education:**

Renal transplant patients are on long-term immunosuppressive medication which may cause gastrointestinal lesions. H pylori infection which is one of the most important and treatable factor leading to a peptic ulcer disease should be treated and eradicated before embarking for transplant.

## Introduction

Although gastrointestinal symptoms are very frequent in kidney transplant patients, there is a paucity of information about the *Helicobacter pylori* Infection (*H.Pylori*) in kidney recipients (1).Recently,*H. Pylori* infection is accepted as an etiological factor of peptic ulcer disease, chronic gastritis, and other gastrointestinal disorders (2). Gastrointestinal symptoms, particularly pyrosis, heartburn and regurgitation are frequent findings, which are known to occur with increased frequency of 20%-60% in kidney transplant recipients (1,2).The frequency of severe complications is about 10% among renal transplant recipients and 10% of those who might prove fatal (1-3). In addition, studies have shown an association between *H. pylori* and gastric carcinoma. *H.Pylori*, is a gram-negative spiral bacillus existing in the mucus layers of the human stomach (2,3). It has now been found that *H. pylori* is one of the co-factors implicated in the progress of neoplastic transformation of gastric mucosa. A particular gastric lymphoma called mucosa-associated lymphoid tissue lymphoma may develop in renal transplant patients, which is generally responds to the eradication of this bacteria (1-3). Considering the previously mentioned data concerning the importance of *H. pylori* infection in kidney patients, at this study, we sought to investigate the association of some demographic and biochemical indices of kidney transplant patients with serum *H. pylori* antibody titer as a sign of *H. pylori* infection.

## Patients and Methods

A cross-sectional study was conducted on a group of renal transplant patients who were referred to the University Clinic for continuing their treatment. All patients signed the consent form for participation in this study. Exclusion criteria was included the presence of any active or chronic infection, acute rejection and intake of antibiotics during the past two months and also taking drugs which were interfered with gastric acid production and function during the past two months, like non-steroidal anti-inflammatory drugs, antacids, proton pump inhibitors and H_2_ receptor antagonists .

After admission, all patients were examined and their medical history, including the time of kidney transplantation and their drugs were obtained. Patients were examined for body mass index (BMI). BMI was calculated using the standard formula (weight in kilograms/height in square meters) (4). The basic immunosuppressive regimen of the recipients was consisted of a combination of cyclosporine at a mean dose of 190±60 mg/d (median of 200 mg/d) and 7.5 mg/d prednisolone for all patients and mycophenolate mofetil in 46% of the patients at a dose of 1500±500 mg/d (median of 1500 mg/d) or azathioprine at a dose of 50 mg/d to 100 mg/d in 26% of the patients.

Serum *H. pylori* IgG antibody titer (titer >10 U/mL positive) was measured as follows by the enzyme-linked immunosorbent assay (ELISA) method using a standard kit. Also, peripheral venous blood samples were collected for biochemical analysis, including serum creatinine, blood urea nitrogen using standard kits after an over-night fast. Creatinine clearance was calculated from creatinine, age and body weight (5).The research followed the tenets of the Declaration of Helsinki and informed consent was obtained. The research was approved by ethical committee of Shahrekord University of Medical Sciences.

The results are expressed as mean±SD and median values. Statistical correlation was calculated using the partial correlation test. For comparison between groups, we used the student's *t*-test.

All statistical analysis was performed with the SPSS 11.5 (SPSS Inc., Chicago, IL, USA). Statistical significance was determined at p<0.05.

## Results

A total of 72 patients including 47 males and 25 females were enrolled in this study. Patients’ man age was 44 ±12 years. The mean BMI of the patients was 44±12.5 kg/m^2^ .The mean time of transplantation was 67.5±42 months (median= 62 months). The mean creatinine clearance was 53±11 mL/min (median = 56 mL/min). The mean of serum *H. pylori* IgG antibody titer was 3±4.6 U/mL (median =1 U/mL) .In this study, there was no significant difference of *H. pylori* IgG antibody serum value between males and females and diabetics or non-diabetics (p>0.05). There was no significant correlation between serum *H. pylori* IgG antibody value and the patients’ age or BMI (p >0.05). A significant positive association of serum *H. pylori* value and creatinine clearance was seen (r =0.26, p=0.02) (adjusted for duration of renal transplantation).

## Discussion

In this study, there was not any significant difference of *H. pylori* IgG antibody levels between males and females. Moreover, no significant relationship between serum *H. pylori* titer and patients’ age was found. A significant positive relation between serum *H. pylori* antibody and creatinine clearance was observed.

Kidney transplantation is the treatment of choice among renal replacement therapies. While, renal transplant patients under immunosuppressive therapy, are susceptible to viral or bacterial infections and various malignancies (1-3,6) Considering the various evidences supporting causal effects of *H. pylori* infection on the development of gastric malignancies, the interaction of *H. pylori* infection and kidney transplantation is of significant importance (1-3,6).

Previous studies have shown an association between *H. pylori* and gastric cancer and in the *H. pylori* (1-3,6). In the study conducted by Kashiwagi *et al*. they found that *H. pylori*-positive titer was accounted for only 23.5% of the renal transplant patients (7). Cocchiara *et al*. performed a study on 61 end-stage renal disease subjected for renal transplantation underwent a gastroscopy to detect *H. pylori.* They divided the 52.4% *H. pylori* -Positive cases into 2 groups: (a) contained 17 patients who were underwent treatment for the eradication of the infection and (b) contained 15 untreated patients (8). They found that, the presence of gastric or duodenal ulcers was significantly higher in the non treated patient than in the treated *H. pylori* -positive patients and significantly higher in the none treated *H. pylori* -positive patients than in the *H. pylori* -negative patients. They concluded that HP-positive patients should therefore be treated for the infection, to elude a long-term significant increase of gastric and/or duodenal peptic disease subsequent to renal transplantation in these immune depressed subjects (8).

## Conclusion

The important point of our investigation was the significant positive association of serum *H. pylori* antibody titer with kidney function in renal transplant patient. It means that with gradual loss of kidney function, the probability of *H. pylori-*infection is increased. While renal transplant patients are on long-term immunosuppressive medication which may cause gastrointestinal lesions, hence, at least H. pylori infection which is one of the most important and treatable factor leading to a peptic ulcer disease should be treated and eradicated before embarking for transplant . However, further investigation is needed to define the clinical significance of our findings.

## Authors’ contributions

HN designed and performed the research. MRK edited the draft. HN prepared the final manuscript.

## Conflict of interests

The author declared no competing interests.

## Ethical considerations

Ethical issues (including plagiarism, data fabrication, double publication) have been completely observed by the author.

## Funding/Support

None.
